# Cartilage regeneration in zebrafish depends on Nrg1/ErbB signaling pathway

**DOI:** 10.3389/fcell.2023.1123299

**Published:** 2023-04-04

**Authors:** Dora Sapède, Sarah Bahraoui, Léa Abou Nassif, Audrey Barthelaix, Marc Mathieu, Christian Jorgensen, Farida Djouad

**Affiliations:** ^1^ IRMB, University Montpellier, INSERM, Montpellier, France; ^2^ CHU Montpellier, Montpellier, France

**Keywords:** cartilage, regeneration, neuregulin 1, chrondrocytes, osteoarthritis

## Abstract

**Objective:** Cartilage, as the majority of adult mammalian tissues, has limited regeneration capacity. Cartilage degradation consecutive to joint injury or aging then leads to irreversible joint damage and diseases. In contrast, several vertebrate species such as the zebrafish have the remarkable capacity to spontaneously regenerate skeletal structures after severe injuries. The objective of our study was to test the regenerative capacity of Meckel’s cartilage (MC) upon mechanical injury in zebrafish and to identify the mechanisms underlying this process.

**Methods and Results:** Cartilage regenerative capacity in zebrafish larvae was investigated after mechanical injuries of the lower jaw MC in *TgBAC(col2a1a:mCherry)*, to visualize the loss and recovery of cartilage. Confocal analysis revealed the formation of new chondrocytes and complete regeneration of MC at 14 days post-injury (dpi) via chondrocyte cell cycle re-entry and proliferation of pre-existing MC chondrocytes near the wound. Through expression analyses, we showed an increase of *nrg1* expression in the regenerating lower jaw, which also expresses Nrg1 receptors, ErbB3 and ErbB2. Pharmacological inhibition of the ErbB pathway and specific knockdown of Nrg1 affected MC regeneration indicating the pivotal role of this pathway for cartilage regeneration. Finally, addition of exogenous NRG1 in an *in vitro* model of osteoarthritic (OA)-like chondrocytes induced by IL1β suggests that Nrg1/ErbB pathway is functional in mammalian chondrocytes and alleviates the increased expression of catabolic markers characteristic of OA-like chondrocytes.

**Conclusion:** Our results show that the Nrg1/ErbB pathway is required for spontaneous cartilage regeneration in zebrafish and is of interest to design new therapeutic approaches to promote cartilage regeneration in mammals.

## Introduction

Cartilage is a connective tissue whose main function is to withstand mechanical loading in the joints. As cartilaginous tissue has limited capacity to repair, traumas and aging often lead to irreversible cartilage erosion and disabling joint pathologies. A challenge for regenerative medicine is to promote the formation of new functional cartilage in patients to avoid the development and/or progression of osteoarticular diseases, alleviate pain and restore movements. Several approaches such as cell therapy have been used to prevent cartilage degradation but, so far, no effective treatment to regrow functional cartilage has been identified. An alternative strategy to counteract cartilage degradations might consist in identifying the mechanisms involved in the regeneration of cartilage in species endowed with this capacity and to propose new therapeutic targets that will enrich drug discovery pipeline.

In contrast to adult mammals, several vertebrate species display robust capacity to regenerate complex structures after amputation or severe injury. For instance, adult zebrafish can regenerate organs such as the brain, liver, heart (Poss et al., 2002; Gemberling et al., 2013; Marques et al., 2019) and skeletal elements such as its caudal fin, skull and jawbone after large-scale lesions (Geurtzen et al., 2014; Paul et al., 2016). At larval stages, caudal fin regeneration is achieved via similar molecular mechanisms as in adult (Kawakami et al., 2004) but regenerative events are more rapid and can be easily manipulated and monitored in real-time due to the small size, permeability and transparency of the young individuals. The zebrafish larva thus offers an advantageous system to study at a high resolution and in a high-throughput manner the mechanistic of regenerative processes for a wide range of tissues or cell types.

In this study, we examine the regenerative ability of cartilage in zebrafish larvae at 3 days post-fertilization (dpf) by performing mechanical wounds of the lower jaw that remove approximately 50% of MC and identify the underlying mechanisms. Together our results revealed that zebrafish larvae exhibit the capacity to regenerate their cartilage after injury through the Nrg1/ErbB pathway.

## Materials and methods

### Zebrafish

Animal experiments using zebrafish were performed at the University of Montpellier according to the European Union guidelines for the handling of laboratory animals and were approved by the Comité d’éthique en expérimentation animale n°036 (approval number: A3417237, reference: APAFIS #32511-2021072114172657 v2).

Zebrafish lines were kept under standard conditions (Nguyen-Chi et al., 2014). Larvae were obtained by natural spawning from pairs or adults then raised at 28.5°C. The zebrafish lines used were as follows: AB wild type zebrafish (ZIRC), *TgBAC(col2a1a:mCherry)* allele hu5910 Tg (Hammond and Schulte-Merker, 2009) abbreviated *Tg(col2a1:mCherry)*, *Tg(sox10:GFP)* allele ba4Tg (Huc-Brandt et al., 2014) and *Tg(Xla.Eef1a1:H2B-Venus)* allele zf499 Tg (Recher et al., 2013).

### Lower jaw injury

Lower jaw injury was performed in 3 dpf larvae anaesthetized with 0.016% Tricaine (MS222, Sigma) diluted in zebrafish water and mounted ventral side up in 1.5% low melting point agarose (LMP agarose, Sigma). Briefly, the lesion was done using a sterile syringe needle (27Gx^3^/_4_‘’(0.4 mm × 19 mm)) under a classical macroscope by cutting only the most anterior part of the lower jaw to obtain a amputation of approximately 50% of the total length of the MC. Accuracy of the lesion was checked in the larvae kept anesthetized and mounted under a MVX10 Olympus macroscope.

### Imaging

Confocal imaging of the growing and regenerating MC in live anesthetized or fixed larvae was done by acquisition of Z-stacks series on inverted confocal microscopes: Leica TCS SP5 and Leica TCS SP8 (Leica Application Suite V3.2 and V3.5, respectively). Images were then analyzed using Fiji Software (ImageJ 1.52p). For time-lapse imaging, fish were mounted in 1% LMP agarose and covered with zebrafish water containing 0.016% Tricaine in 35 mm glass-bottom dishes sealed with parafilm to avoid drying.

### BrdU incorporations

Uninjured and injured larvae were transferred in 10 mM bromodeoxyuridine (BrdU, Sigma, Catalog #: 59-14-3) diluted in zebrafish water containing 1.5% DMSO and incubated for 4, 8, or 16 h at 28.5°C. For pulse-fix experiments, larvae were rinsed three times in fresh zebrafish water, then immediately anesthetized and fixed in 4% PFA in PBS for 2 h at room temperature or overnight at 4°C. For pulse-chase experiments, larvae were allowed to recover in fresh zebrafish water until 6 dpf/72 hpi after BrdU incorporations and rinsing. Fish were finally dehydrated in ethanol and stored at −20°C and processed for immunohistochemistry.

### Immunofluorescence and *in situ* hybridization

BrdU immunohistochemistry were performed as previously described (Ma et al., 2008) with the following modifications for double immunostainings: fish were rinsed, permeabilized and processed for blocking then incubated overnight at 4°C in blocking solution containing two primary antibodies: Anti-DsRed rabbit (Living Colors^®^ DsRed Polyclonal Antibody, Catalog #: 632,496) at 1:500, Anti-BrdU from mouse IgG1 (Roche, Catalog #: 11170376001) at 1:100. After extensive washes at room temperature, the larvae were incubated in blocking solution containing a mix of secondary antibodies: Goat anti-Rabbit IgG Alexa Fluor Plus 488 (Thermofisher, Catalog #: A32731) and Goat anti-Mouse IgG, Alexa Fluor Plus 594 (Thermofisher, Catalog #: A32742) diluted at 1:500.

To perform whole mount ISH, larvae were fixed at desired stages in 4% PFA in PBS overnight at 4°C then processed through standard protocol (Nguyen-Chi et al., 2015).

### Pharmacological treatment

Larvae were treated with 3.75 μM PD168393 (Sigma, catalog #: 194,423-15-9) in zebrafish water containing 0.2% DMSO immediately after injury and until 5 dpf/48 hpi. Control larvae were incubated in zebrafish water containing 0.2% DMSO for the same period of time. Fish were then rinsed three times for 5 min in fresh zebrafish water and let to recover until 6 dpf/72 hpi before being anesthetized and mounted for *in vivo* confocal imaging.

### Mosaic morpholino injections

Early embryos were injected either with 1 nL of a mix containing Mo-*nrg1* (final concentration: 300 μM, sequence: 5′-GCT​GCT​GCA​CAG​AGG​AAC​ACA​AC-3’; Gene Tools) and green fluorescent dextran (OG; Oregon Green) or with OG alone at 8 to 16-cell stage. We then selected at 3 dpf the fish that displayed OG fluorescence at the level of the skin covering the lower jaw, which corresponds to the region of injury-induced *nrg1* expression, and performed amputations. Fish were let to recover until 6 dpf/72 hpi before being anesthetized and mounted for *in vivo* confocal imaging.

### Cell counting

For topological mapping, three main zones of interest were defined on the confocal z-stacks analyzed: the anterior zone (100 µm, centered on the tip of MC), the joints (50 µm × 50 µm square containing the entire head of Meckel’s cartilage) and the lateral parts of MC. The number of cells was then quantified manually using Fiji software to mark the cells.

### Murine articular chondrocyte culture

3-day-old Swiss mouse knees and femoral heads were dissected as previously described (Gosset et al., 2008). In 12-well culture plates from TPP (Techno Plastic Products, Switzerland), chondrocytes were plated and cultured for 5 days within a defined proliferative medium. After 5 days of culture, murine chondrocytes were exposed to a degenerative stimulus mimicking osteoarthritic-like changes induced by 1 ng/mL IL-1β (R&D Systems) for 24 h and then treated or not with 50 ng/mL NRG1 (Recombinant Human Neuregulin-1, NRG1, R&D Systems) before recovering chondrocytes for RT-qPCR analysis 3 days after.

### RT-qPCR analyses

Total RNA was isolated from zebrafish or mouse chondrocytes using the RNeasy kit and according to the supplier’s protocol (Qiagen, Courtaboeuf). Using 100 U of M-MLV reverse transcriptase (Bioline) we reverse transcribed total RNA (0.5 μg) and performed PCR reactions as previously described (Maumus et al., 2013). Primer sequences (SYBR Green Technologies) can be provided upon request. All values were normalized to the Rps9 or ef*1a* housekeeping genes, for mouse and zebrafish, respectively, and expressed as relative expression using the respective formulae: 2^−ΔΔCT^.

cDNA was synthesized by reverse transcribing 500 ng of RNA into cDNA using the SensiFAST™ cDNA Synthesis Kit (Bioline, Meridian Life Science^©^ Company). Quantitative PCR was performed on 6.25 ng or 12,5 ng of cDNA, for mouse and zebrafish, respectively, using the SensiFAST™ SYBR^®^ No-ROX kit (Bioline, Meridian Life Science^©^ Company).

### Statistical analyses

Unpaired Mann-Whitney tests were performed using GraphPad Prism 6 software (San Diego, CA, United States) to test the significance of the data presented in this study.

## Results

### Meckel’s cartilage (MC) fully regenerates in larval zebrafish

To address the regenerative capacity of MC in the zebrafish larva, we performed mechanical amputations of the anterior part of the lower jaw at 3 dpf. To confirm the efficiency of our injury protocol and to further monitor *in vivo* the regenerative capacity of MC, we used *TgBAC(col2a1a:mCherry) (* Mitchell et al., 2013) transgenic larvae, herein abbreviated as *Tg(col2a1:mCherry),* in which all the chondrocytes are labelled by a red fluorescent tag. The fish in which 30%–60% of the *col2a1:mCherry* ^+^ fluorescent MC were lost in the lower jaw were selected as successfully injured (average length of injured MC at T0 is 53.48% of the uninjured: Uninjured: 275.6 ± 20.0 µm; Injured: 147.4 ± 22.1 µm).

**FIGURE 1:**
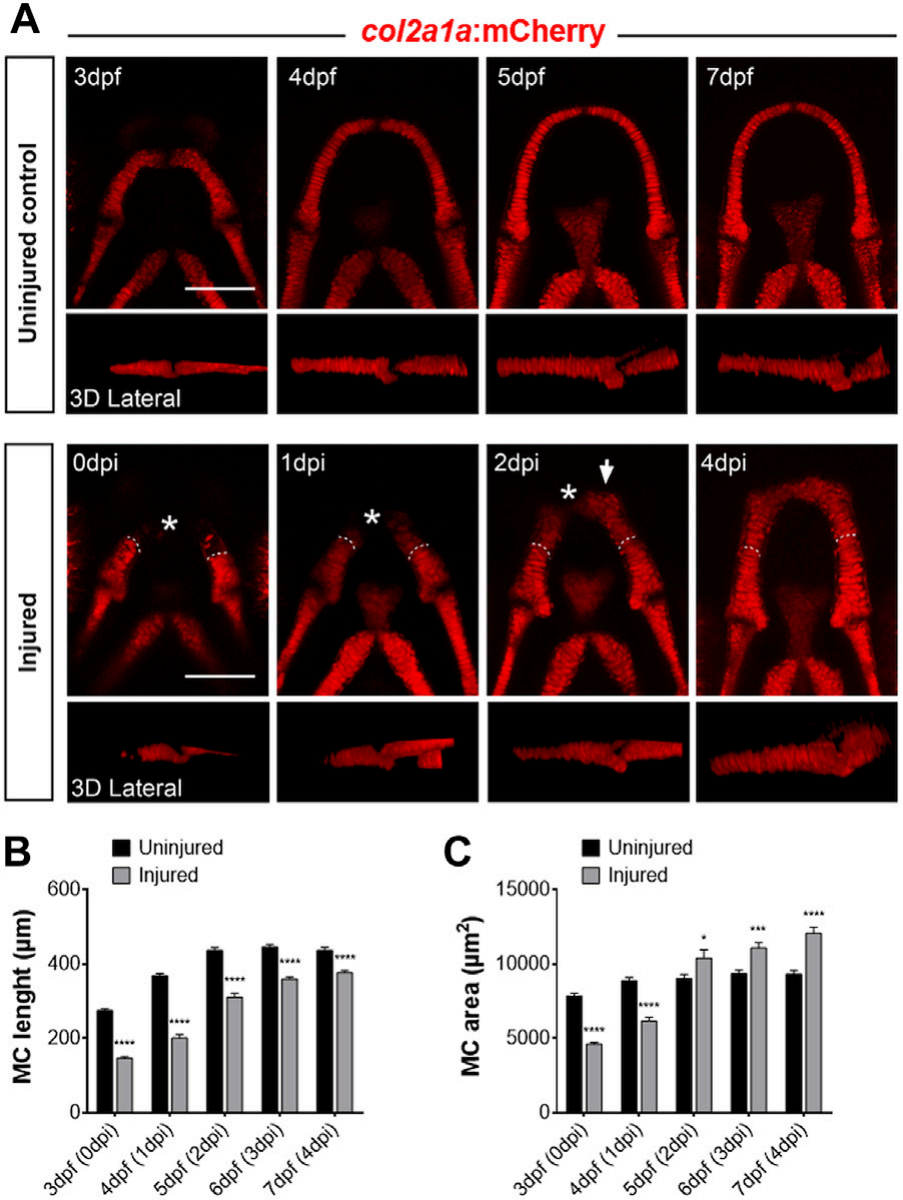
Regrowth of the Meckel’s cartilage (MC) following mechanical injury of the lower jaw in *Tg(col2a1:mCherry)* line **(A)** Confocal analysis of the normal growth of MC between 3 and 7 dpf (upper panel) and formation of new chondrocytes (lower panel) at equivalent stages after surgical amputation of the anterior half of the MC at 3 dpf. The upper row of each panel are maximal projections of z-stacks in ventral views, whereas the lower rows are 3D reconstructions corresponding to each ventral view, oriented in lateral views of the MC. The asterisk marks the amputated anterior part of MC and the white dotted line indicates the site of amputation. Note that a group of new chondrocytes are visible at the tip of the cut MC at 2 dpi (white arrow). **(B)** Time course of MC elongation after injury and comparison with normal growth in age-matched controls from 3 to 7 dpf. **(C)** Time course of MC area increase after injury and comparison with normal growth in age-matched controls from 3 to 7 dpf. Scale bar: 100 µm. Graphs indicate the means ± SEM. N = 10 individuals minimum were analyzed for each condition. Mann-Whitney test was used to compare the length and area in injured and uninjured individuals. *: *p* < 0.05; **: *p* < 0.01; ***: *p* < 0.001; ****: *p* < 0.0001.

Confocal analysis of injured and uninjured *Tg(col2a1:mCherry)* larvae at different time points during 4 dpi allowed us to quantify the recovery of mCherry fluorescence in injured fish. At 2 dpi, low *mCherry* ^*+*^ cells were detected at the anterior end of each lesioned MC branch (Figure 1A, white arrow) indicating that new chondrocytes have formed. Detailed observations of the regrowth dynamics distinguishing the “intact” and “regenerated” parts (Supplementary Figure S1) suggest that while the intact part elongate progressively mainly by chondrocyte division, the “regenerated” part grows rapidly and massively at the amputation site between 24 and 48 hpi.

From 3-4 dpi, injured cartilage has further elongated and, in most of the cases, the two cut parts have fused together at their anterior extremities. At 4 dpi, injured MC have grown up to 86.2% of the control average length (Uninjured: 437.3 ± 34.7 µm; Injured: 376.8 ± 30.0 µm) and 129.4% of the control average area (Uninjured: 9,331 ± 1,116 µm^2^; Injured: 12,074 ± 1,994 µm^2^), showing that the resulting cartilage is significantly shorter and wider than uninjured MC at an equivalent stage (Figures 1B, C). In addition, injured MC displayed complete fusion of the two lateral *mCherry* ^*+*^ parts suggesting that mandibular symphysis is absent at this stage (compare 4 dpi and 7 dpf; Figure 1A).

**FIGURE 2:**
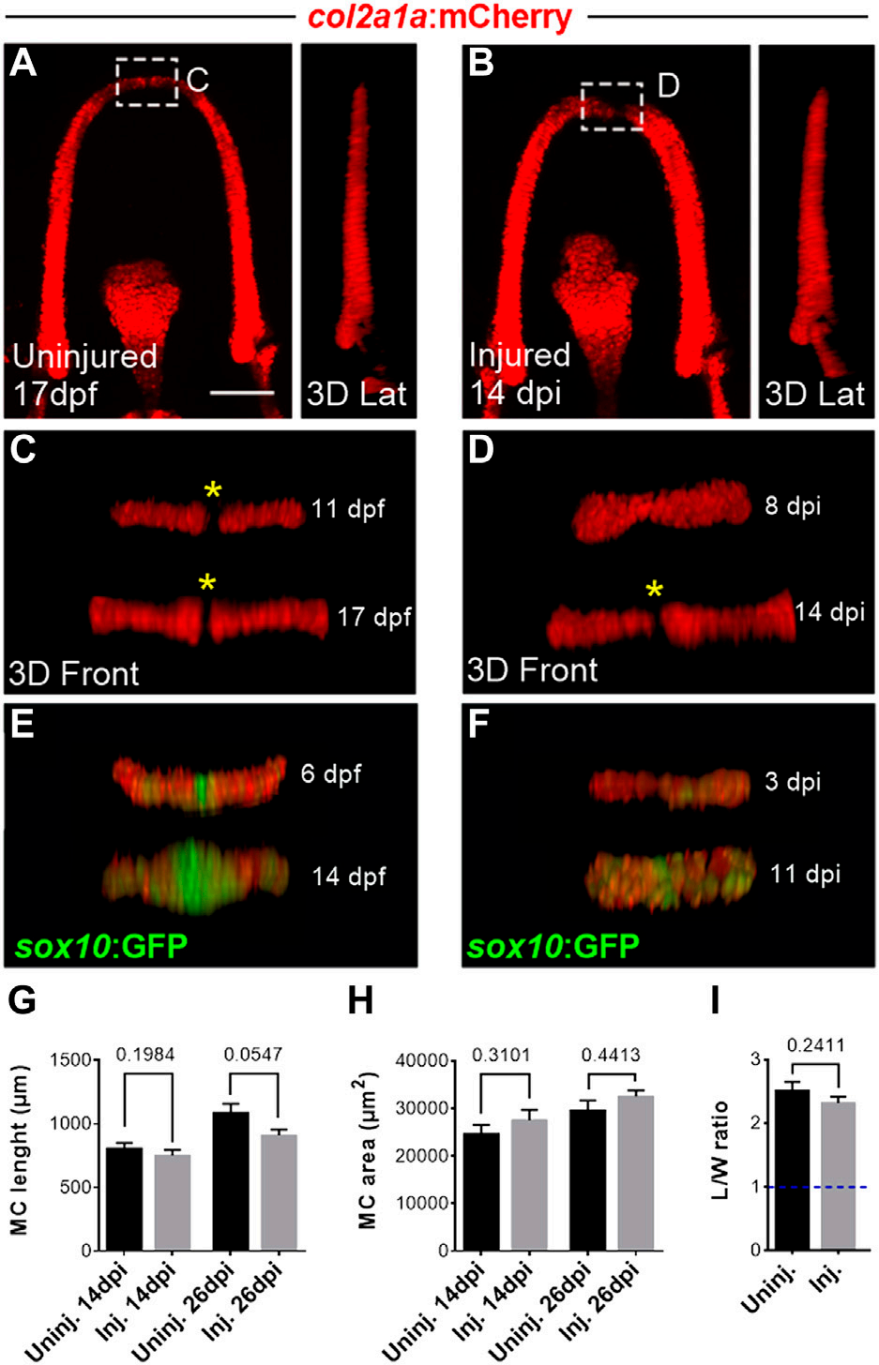
Late observations of MC at 14 and 26 dpi indicate full regeneration of injured cartilage **(A, B)** Confocal maximal projections of z-stacks in ventral views and corresponding lateral 3D reconstructions of the uninjured **(A)** and injured **(B)** MC at 17 dpf (14 dpi). **(C, D)** 3D reconstructions of the distal tip of the uninjured **(C)** and injured **(D)** MC at 11 dpf (8 dpi) and 17 dpf (14 dpi). The yellow asterisks mark the absence of *col2a1:mCherry* expression at the level of the central cells of the distal tip until late stages of development in uninjured controls. Note the absence of *col2a1* fluorescence in the central cells prefiguring the mandibular symphysis, whereas the regenerated MC harbors a continuum of cells expressing *col2a1* at 8 dpi. Later on, central cells at the tip had lost *col2a* expression at 14 dpi. **(E, F)** 3D reconstructions of the distal tip of the uninjured **(C)** and injured **(D)** MC at 6 dpf (3 dpi) and 14 dpf (11 dpi) in the double *Tg(sox10:GFP;col2a1:mCherry) line*. The central cells of the distal tip contains GFP expressing cells during development in uninjured controls. Note that the regenerated MC harbors a continuum of cells expressing low levels of GFP at 3 dpi but higher expression at the tip at 11 dpi. **(G, H)** Graphs comparing MC length **(G)** and area **(H)** in injured versus uninjured fish at 14 and 26 dpi. N = 11 to 22 individuals for each condition. **(I)** Graphs comparing the L/W ratio of MC *col2a1* ^*+*^ cells within the 100 µm region located at the anterior tip of MC in injured versus uninjured fish at 14 dpi. Note that the *col2a1* ^*+*^ cells are similarly elongated (the dotted blue line indicates a ratio of 1 which would correspond to round cells. Scale bar: 100 µm. Graphs indicate the means ± SEM. N = 50 cells were analyzed in uninjured, n = 70 cells in injured for the graph in I. Mann-Whitney test was used to compare the MC lenght and area and the L/W ratio of cells in injured and uninjured individuals. *p* > 0.05.

In contrast to repair processes that may replace some lost tissue with structural changes and functional loss, regeneration implies the recovery of the mass, cell type composition and organization, and of the function of the original structure (Londono et al., 2018). To determine whether the new cartilage produced after mechanical injury in zebrafish is dysmorphic, we performed later observations at 8, 11, 14 and 26 dpi and compared these data with age-matched controls. Quantification of MC length and area showed that injured MC recovered proportions similar to controls from 14 dpi (Figures 2A, B, G, H). The roundness (L/W ratio) of *col2a1* ^+^ chondrocytes located at the most anterior position in the MC were also similar between injured and uninjured at 14 dpi/17 dpf, suggesting that the cells recover at this stage an elongated shape in the regrown area, similar to control cells that are stacked (Figure 2I). Observations of cell organization at these later stages revealed in addition that a gap in *mcherry* ^*+*^ fluorescence is again found from 14 dpi at the most anterior portion of MC (Figures 2C, D), a region in which only *sox10* expressing cells are found in control uninjured MC (Figures 2E, F), suggesting *de novo* formation of the mandibular symphysis at this stage.

In summary, quantitative and qualitative analysis of MC regrowth after amputation led us to conclude that MC can fully regenerate in zebrafish larvae after surgical amputation.

### Injury triggers cell cycle re-entry of MC chondrocytes

**FIGURE 3:**
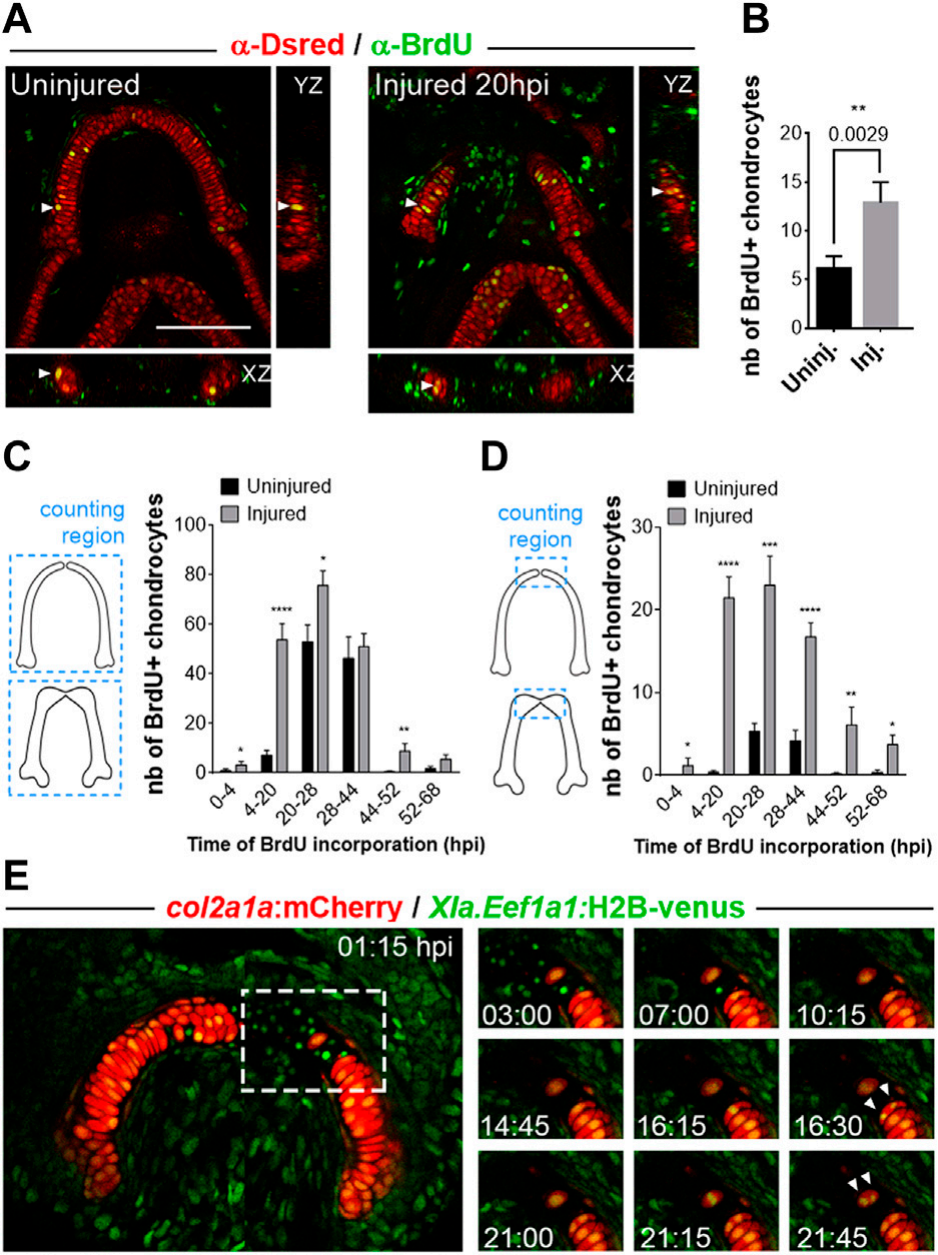
Analyses of cell proliferation in growing versus regenerating MC in *Tg(col2a1:mCherry)* line **(A)** Double immuno-stainings against BrdU (green) and Ds-Red (red) after BrdU incorporations performed during 16 h between 4 and 20 hpi. The pictures are confocal maximal projections of z-stacks in ventral views of MC in injured at 20hpi versus age-matched uninjured fish. The white arrowheads mark the BrdU^+^ chondrocytes highlighted in the orthogonal views. **(B)** Countings of double BrdU^+^/mCherry^+^ cells in injured MC at 20hpi (immediately after a 16 h pulse at 4–20 hpi) versus uninjured MC at an equivalent stage. **(C, D)** Countings of double BrdU^+^/mCherry^+^ cells in injured MC at 3 dpi after sequential periods of BrdU incorporations in pulse-chase strategy. The double positive cells counted in graph **(C)** correspond to cells found in each MC half, whereas the cells counted in **(D)** are double positive cells mapped at 3 dpi in the anterior tip of each MC half. **(E)** Time lapse analysis of chondrocyte cell division at the wound in a double transgenic *Tg(col2a1:mCherry;XIa.Eef1a1:H2B-venus)* fish taken from 1 to 22hpi. The panel on the right describes the successive steps of division of two chondrocytes (white arrowheads) located at the border of the wound at the anterior end of cut MC (dotted white line in the left picture). Scale bar: 100 µm. Graphs indicate the means ± SEM. Mann-Whitney test was used to compare the number of BrdU+ cells in injured and uninjured individuals. *: *p* < 0.05; **: *p* < 0.01; ***: *p* < 0.001; ****: *p* < 0.0001.

We then sought to examine the mechanisms involved in the regeneration of MC and to test whether cell proliferation dynamics change in the MC upon injury to compensate the lost tissue. To test this hypothesis, we used incorporations of BrdU in pulses for different time periods during 3 dpi and compared the number of double labeled MC cells between injured and uninjured fish with anti-DsRed and anti-BrdU stainings. This experiment revealed that the number of chondrocytes entering S-phase increases significantly within the first hours following injury (4–20 hpi; Figures 3A, B) as compared to controls. The presence of doublets of chondrocytes labelled with anti-BrdU in the injured cartilage suggests that chondrocytes have already divided at 20 hpi, whereas they did not in the uninjured age-matched control (Figure 3A). These results indicate that injury triggers cell cycle re-entry of MC chondrocytes, that contribute to the regrowth of this structure.

To better characterize the timing and pattern of new chondrocyte formation, we used BrdU pulse-chase strategy. Double anti-DsRed and anti-BrdU immunostainings performed at 3 dpi/6 dpf after sequential periods of BrdU incorporations during regeneration showed a significant increase in the number of labelled chondrocytes in regenerating MC compared with controls (Figure 3C). Our results also indicate that a high number of new chondrocytes present at 3 dpi are generated during the first 28 h following injury. Detailed mapping of the BrdU/DsRed double positive cells located in joints, lateral and anterior parts of the regenerating MC at 3 dpi showed that the increased production of chondrocytes is sustained at the anterior end of regenerated MC (Figure 3D), a region which is scarcely labelled in uninjured control fish at the different stages examined. In contrast, we found only a moderate increase at 3 dpi in the production of chondrocytes in the other regions of the injured MC (data not shown). Together, these results suggest that injury triggers both a global increase in cell proliferation in MC and the local addition of new chondrocytes at the anterior ends of the wounded mandibular cartilage.

### Wound-associated chondrocytes actively divide during regeneration

To test whether the new chondrocytes that accumulate preferentially at the site of injury originate from pre-existing chondrocytes, we analyzed chondrocyte proliferative behavior in real time during the first 2 days of regeneration. To this end we used double *Tg(col2a1:mcher*ry; *Xla.Eef1a1:H2B-Venus)* transgenic fish in which all cell nuclei are labelled in green allowing us to follow chondrocyte cell divisions by *in vivo* video time lapse confocal microscopy. A movie taken immediately after lower jaw injury (Figure 3E) showed that two chondrocytes located at the wound border divide during the first 22 h of regeneration. Closer examination of cell behaviors in the double transgenics indicates that numerous cell nuclei visible in the wounded zone, that most probably correspond to chondrocytes damaged during injury, are eliminated by apoptosis during the first hours post-injury before cell division occurs. Time lapses performed at later stages showed a similar pattern of chondrocyte division (data not sown), indicating that wound-associated chondrocytes divide actively during the regenerative process for an extended period of time.

### Contribution of immature chondrocytes during cartilage regeneration

The *Tg(sox10:GFP)* line labels the migrating neural crest cells (NCC) at early embryonic stages (Dutton et al., 2008). Expression of GFP is reactivated from 48hpf in the differentiating chondrocytes of the jaw, which are NCC cranial skeletal derivatives, and this expression persists for several days (Dutton et al., 2008; Paul et al., 2016; Giovannone et al., 2019; Smeeton et al., 2021). A previous study has determined that distinct populations of chondrocytes can be classified by their relative expression of *col2a1:mCherry* and *sox10:GFP* transgenes. First, *sox10+*/*col2a1-*cells located at the level of the jaw joint and mandibular symphysis are chondrocytes precursors (Brunt et al., 2016) that proliferate, migrate and differentiate to ensure post-embryonic growth of MC and jaw joint (Brunt et al., 2017). Then, *sox10* ^*+ high*^ */col2a1* ^*+*low^chondrocytes located at the joints are articular chondrocytes that maintain low levels of mature chondrocyte markers.

**FIGURE 4:**
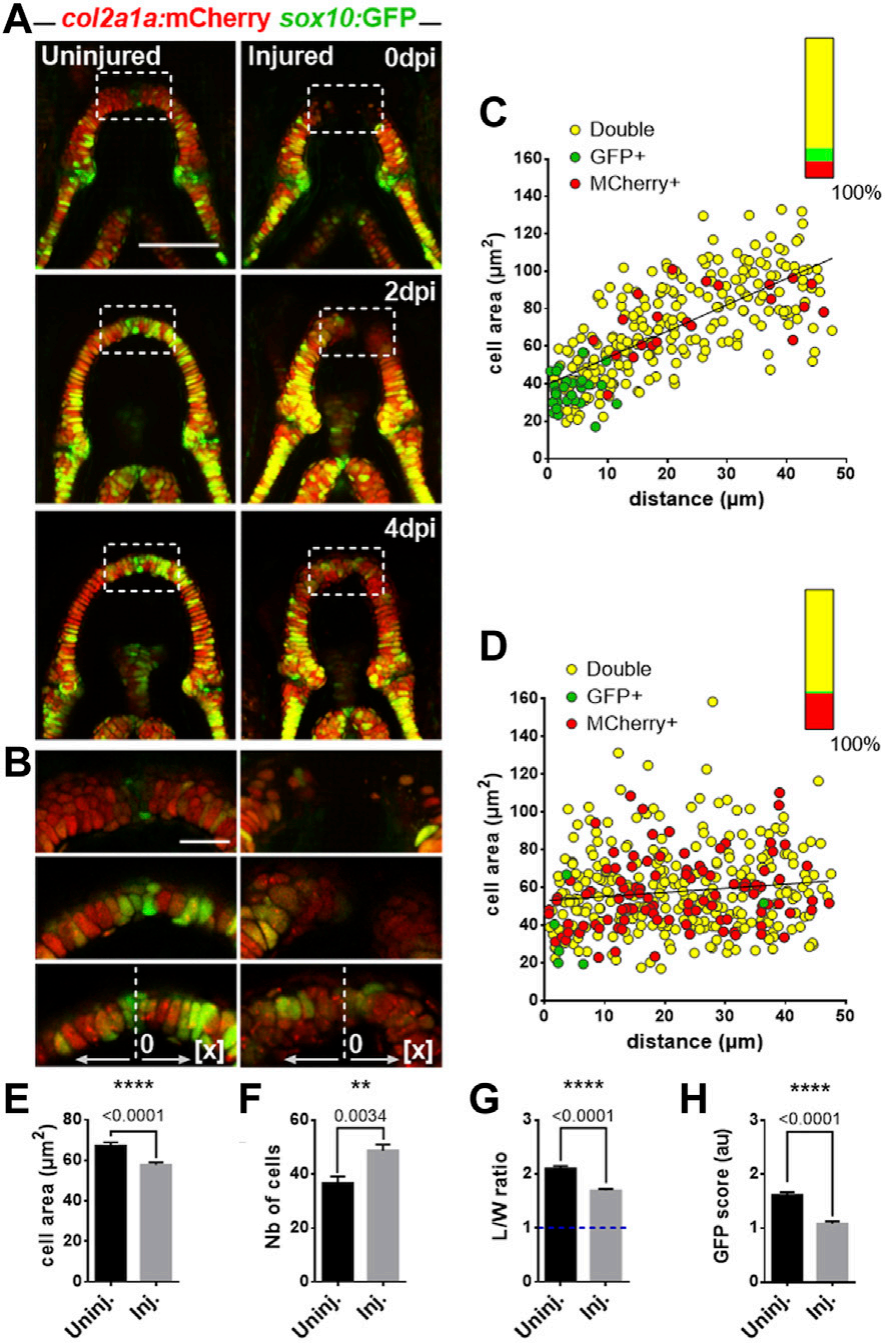
Distribution of cells expressing distinct levels of *sox10:GFP* and *col2a1:mcherry* transgenes during MC regeneration **(A)** Confocal maximal projections of MC in ventral views for control uninjured and injured fish at 0, 2 and 4 dpi. **(B)** Close-up on the midzones of the control uninjured and injured lower jaws presented in A at 0, 2 and 4 dpi (delineated by the white dotted line). The center of the midzone is indicated by a dotted line that represents the “0” of the *x*-axis for mapping the cells. **(C, D)** Mapping of the cell areas depending on their distance to the center of the corresponding MC in injured **(D)** versus uninjured **(C)**. Data from the two hemi-segments of each MC are placed in the same direction for analyses. Qualitative data about the expression of *sox10:GFP* and *col2a1:mcherry* transgenes are color coded on the graph (red: *col2a1:mcherry* ^*+*^ only, green: *sox10:GFP* ^*+*^ only, *yellow:* double positive *for sox10:GFP* and *col2a1:mcherry* ^*+*^ *).* A graph on each upper right corner indicates the proportions of each cell population. **(E–H)** Graphs comparing different parameters of the cells present in injured versus uninjured MC midzones at 4 dpi. The scoring for fluorescence intensities were as follows: 0 = no fluorescence, 1 = detectable fluorescence, 2 = clear fluorescence, 3 = bright fluorescence. Scale bar: 100 µm **(A)**; 25 µm **(B)**. Four individuals of each condition were analyzed for each experiment, 2 independent experiments (n = 8), in total 293 and 390 cells were analyzed for uninjured and injured condition, respectively. Graphs indicate the means ± SEM. Mann-Whitney test was used to compare the indicated parameters in injured and uninjured individuals. *: *p* < 0.05; **: *p* < 0.01; ***: *p* < 0.001; ****: *p* < 0.0001.

Upon injury at 3 dpf, the *sox10* ^*+*^/*col2a1* ^*-*^ precursors located at the level of the mandibular symphysis have been removed suggesting that other chondrocytes might reactivate proliferative abilities upon injury (Figures 4A, B). To address the potential contribution of the immature versus fully differentiated chondrocytes during the regenerative process, we crossed *Tg(col2a1:mCherry)* and *Tg(Sox10:GFP)* zebrafish and examine their relative proportion in regenerating MC. From 3 to 7 dpf, *mCherry* ^*+*^ MC chondrocytes in the double transgenics express variable levels of GFP (Figures 4A, B) suggesting heterogeneous levels of differentiation within the developing cartilage. At 4 dpi, we observed that the anterior part of the regenerated MC is almost devoid of cells expressing high levels of GFP (Figures 4B, D, H; average GFP score: 1.62 ± 0.91 in uninjured control versus 1.09 ± 0.82 in injured), indicating that the pool of *sox10* ^*+ high*^ chondrocytes present in the distal part of injured MC get exhausted during regeneration.

The anterior part of the MC during normal growth has been defined as the midzone, a zone in which *col2a1:mCherry* ^*+*^ cells initially form a disorganized group of small and round cells at 3 dpf, that progressively grow, differentiate and stack to integrate the growing MC (Ling et al., 2017). Here, during MC regeneration, we observed that the anterior part of the regenerated tissue resembles the original midzone of MC at 3 dpf as it contains significantly more cells than the age-matched control (Figure 4F in 100 µm length, uninjured: 36.63 ± 7.09 versus 48.75 ± 6.27 in injured). These cells are smaller (Figure 4E average cell area: 67.31 ± 25.82 µm^2^ in uninjured versus 57.89 ± 21.47 µm^2^ in injured) and rounder (Figure 4G average L/W ratio = 2.11 ± 0.75 in uninjured, versus 1.70 ± 0.54 in injured) than the chondrocytes present in the midzone of age-matched controls.

Mapping of the cell areas relative to the distance to the tip of the MC further showed that the cells located in the midzone at 4 dpi in regenerating MC are not arranged linearly with smaller cells closer to the tip and larger cells farther as in controls (Figures 4C, D). When combined with the data of *col2a1/sox10* expression, mapping analysis confirm that cells expressing *col2a1* only are present in higher proportion in the midzone of newly formed cartilage at all positions. Meanwhile, a population of small cells that express only *sox10*, although highly reduced compared with controls, is also found in injured MC almost exclusively near the tip of MC. This latter result suggests that the population of immature chondrocytes at the mandibular symphysis is reconstituting at 4 dpi.

Together, these results lead us to conclude that the pool of less mature chondrocytes present in the injured cartilage get exhausted during regeneration. *col2a1* ^*+*^/*sox10* ^*+*^^high^ cells might proliferate to give a group of small and poorly differentiated chondrocytes resembling the midzone present at an earlier stage of development that will ultimately replace the lost anterior MC by differentiation and reorganization.

### Role of Nrg1/ErbB pathway in cartilage regeneration

**FIGURE 5:**
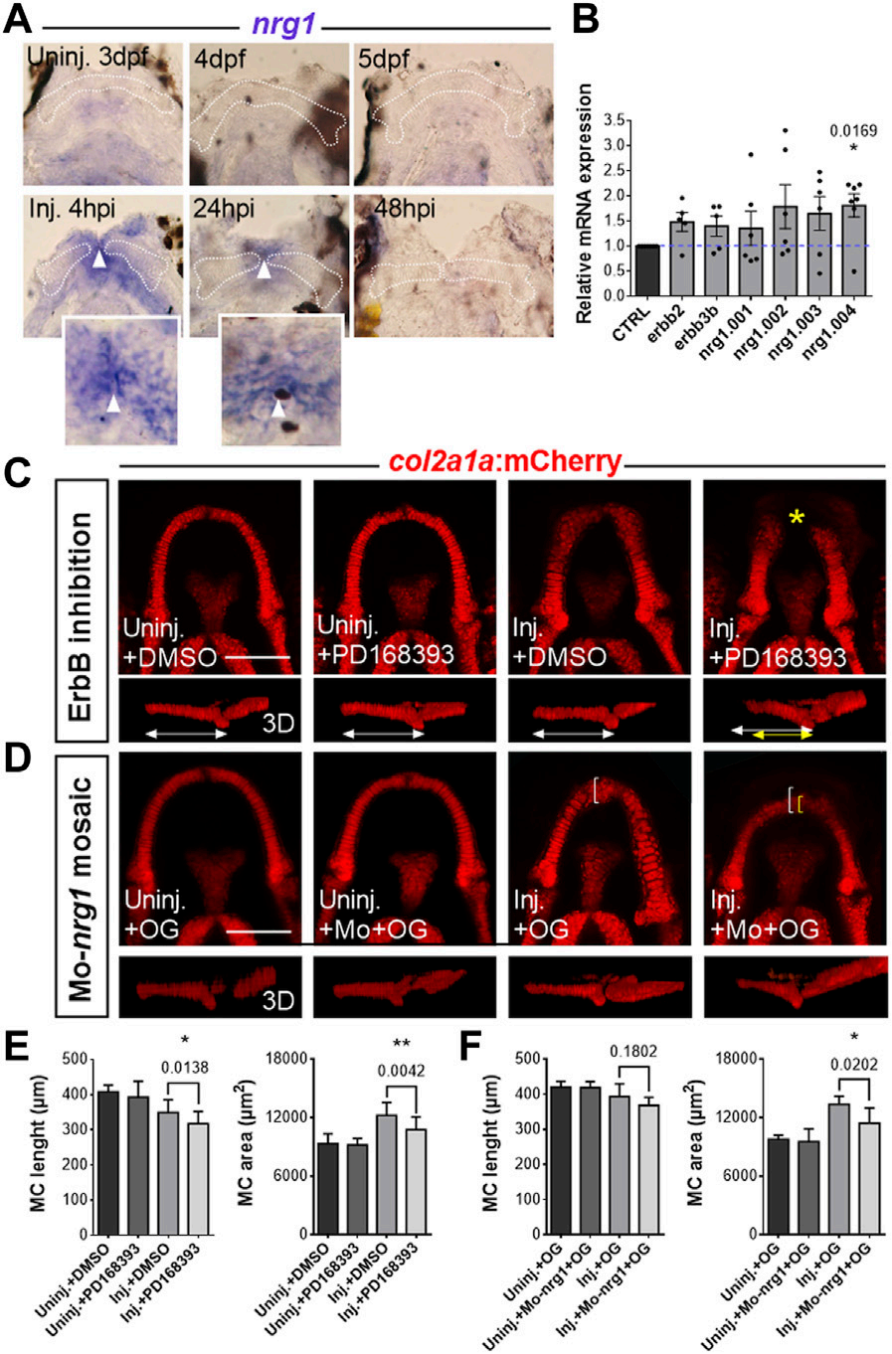
Role of the Nrg1/ErbB signaling pathway in MC regeneration **(A)** Expression analysis of *nrg1* by *in situ* hybridization in fish injured at 3 dpf at the different times post-injury indicated (lower panel) and in uninjured control at equivalent stages (upper panel). MC is delineated by a white dotted line. Insert present a close-up on cells expressing *nrg1* at the level of the epithelium covering the wound below the pictures with positive signal (superficial focal plane). White arrowheads point to the midline of the lower jaw at which the epithelial cells from the two sides have joined. **(B)** Levels of mRNA expression of the genes indicated in injured fish at 24 hpi relative to expression in control uninjured larvae at corresponding stages. N = 5–7 for each gene, all the values were normalized relative to the corresponding control uninjured value. **(C)** Representative pictures of growing uninjured MC and regenerating MC at 3 dpi after incubations of the fish with the pan-ErbB inhibitor PD168393, or with DMSO alone. The treated and untreated MC are presented in ventral views (confocal maximal projections of z-stacks) and lateral views of 3D reconstructed MC. **(D)** Representative pictures of growing uninjured MC and regenerating MC at 3 dpi after mosaic injections of OG with or without Mo-*nrg1.* The fish are presented in ventral views (confocal maximal projections of z-stacks) and lateral views of 3D reconstructed MC. **(E)** Graphs comparing the length and area of uninjured and injured MC, treated with PD168393 or DMSO alone. N = 17-20 for each condition, data from three independent experiments. **(F)** Graphs comparing the length and area of uninjured and injured MC, co-injected with OG and Mo-nrg1 or injected with OG alone. N = 6–11 for each injured condition, data from two independent experiments. Scale bars: 100 µm. Graphs indicate the means ± SD. Mann-Whitney test was used to compare the data obtained in two conditions. *p* values are indicated on the graph when significant. *: *p* < 0.05; **: *p* < 0.01.

To further examine the molecular mechanisms required for MC regeneration, we took a candidate approach. Neuregulin 1 (Nrg1) is a growth factor of the EGF family that has been involved in regeneration in different species and contexts (Bersell et al., 2009; Fricker and Bennett, 2011; Laplace-Builhe et al., 2021). To study a potential role of the Nrg1/ErbB pathway in our model, we first performed an expression analysis of *nrg1* by *in situ* hybridization at different stages in injured and control jaws (Figure 5A). These experiments showed that *nrg1* transcript is strongly induced in the wounded region at 4 hpi. This induction of *nrg1* persists through 24 hpi at the wound region and expression returns to control undetectable levels at 48 hpi, whereas uninjured age-matched control fish display very faint to no staining in their lower jaw at the corresponding stages. Detailed observation of the stained mandibles showed that *nrg1* is expressed mainly in the epithelium covering the wound at 4 hpi and in between the two extremities of injured MC at 24 hpi (inserts in panel A, pictures taken at a superficial focal plane). Although resolution of *in situ* hybridization did not allow us to conclude for the chondrocytes abutting the wound, it showed that *nrg1* is present in tissues adjacent to the wounded cartilage during the first 24 hours following injury.

Nrg1 is present in different isoforms that are coded by alternative splicing of the *nrg1* gene and specific isoforms have distinct biological activities in a transmembrane or paracrine fashion (Kerber et al., 2003; Gambarotta et al., 2014; El Soury and Gambarotta, 2019). Nrg1 ligand, upon binding to ErbB3 or ErbB4, triggers the formation of active homo- or hetero-dimers of ErbB receptors that will control dowstream signaling pathways responsible for cell survival, differentiation and proliferation. Previous studies have shown that ErbB2/3 is required for the regeneration of the zebrafish larval caudal fin (Rojas-Munoz et al., 2009; Laplace-Builhe et al., 2021). RTqPCR performed at 24 hpi and in age-matched uninjured controls confirmed the expression of four splice variants of *nrg1* and of *erbb2* and *erbb3b* mRNA during MC regeneration (Figure 5B). Moreover, the analysis of the expression profile of the *nrg1* splice variants described in zebrafish (i.e., *nrg1.001, nrg1.002, nrg1.003* and *nrg1.004*; ZFIN.org) revealed similar amounts of *nrg1.001, nrg1.002* and *nrg1.003* transcripts in intact and injured tissues, whereas *nrg1.004* expression is significantly increased at 24 hpi as compared to uninjured controls (Figure 5B). Regarding the expression profile of e*rbb2* and *erbb3b*, we found a trend toward an increase for both receptors 24 hpi **(** Figure 5B). Together, these results are coherent with a role of Nrg1/ErbB2-3 pathway in the early steps of MC regeneration.

To study the potential implication of ErbB signaling in cartilage regeneration, we incubated *Tg(col2a1:mCherry)* injured fish with the ErbB inhibitor PD168393 or with vehicle (DMSO) alone and compared the lenght and area of MC regenerates at 3 dpi in these different conditions. This analysis showed that PD168393-treated injured zebrafish display a significant reduction of MC length and area at 3 dpi as compared with injured fish incubated with DMSO alone (Figures 5C, E).

To investigate the specific role of Nrg1 in this process, we performed mosaic injections with a morpholino directed against *nrg1* (Mo-*nrg1*). Injections of Mo-*nrg1* at the one-cell stage resulted in a high variability of gene inactivation with frequent malformations that precluded the study of lower jaw regeneration. To bypass the deleterious effects of systemic morpholino injections on general development and to minimize its dilution to suboptimal levels by successive cell divisions between 3 and 6 dpf, we performed morpholino injections in one cell at the 8- to 16-cell stage. We combined the Mo-*nrg1* with a green fluorescent dextran (OG; Oregon Green) to track the cells that have received the morpholino at the time of amputation. We then selected the fish that displayed OG fluorescence at the level of the skin in the mandibular region, corresponding to the region of strong injury-induced *nrg1* expression, and performed MC amputations. The fish injected with Mo-*nrg1* exhibited defective cartilage regeneration as compared with those that only received the green dextran in the same region (Figures 5D, F). Although the defects were less severe than those obtained with the pan-ErbB inhibitor, this result points to a specific role of Nrg1 in cartilage regeneration.

Altogether, these results suggest that Nrg1/ErbB signaling is required for MC regeneration.

### Nrg1-ErbB signaling activation protects mammalian chondrocytes from IL1β-induced loss of mature chondrocyte phenotype.

**FIGURE 6:**
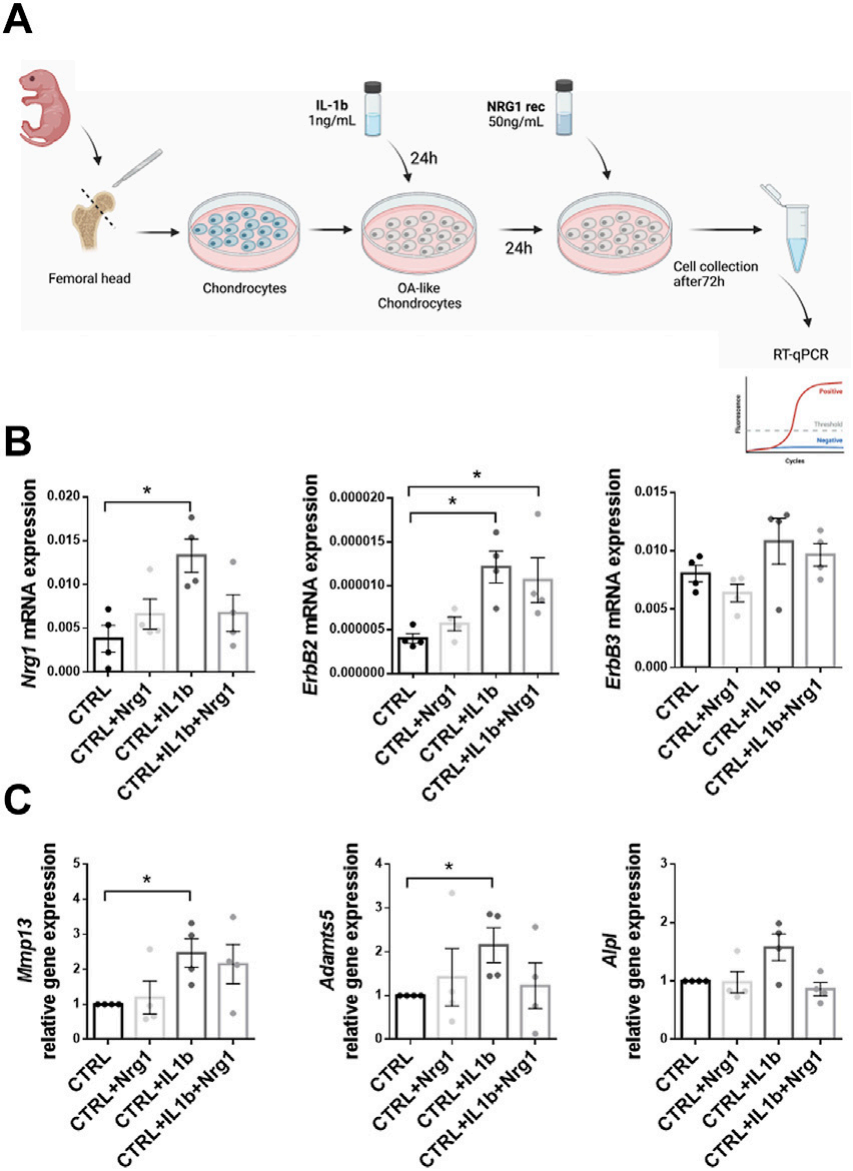
Effect of NRG1 treatment on chondrocytes undergoing an IL1β-dependent degeneration associated with osteoarthritic (OA)-like phenotype *in vitro* **(A)** Design of the experiment. **(B)** *Nrg1*, *ErbB2* and *ErbB3* mRNA expression in naïve chondrocytes (CTRL, not treated with IL1β) and chondrocytes impaired by IL1β, treated or not by NRG1. **(C)** RT-qPCR analysis of CTRL and OA-like chondrocytes (IL1β). Expression profile of hypertrophic (*Mmp13*, *Adamts5* and *Alpl*) marker genes. Each dot represents one biological replicate, and results are expressed as the mean ± SEM. Mann-Whitney test was used to compare control untreated samples and samples treated with either IL1β or IL1β and NRG1. *: *p* < 0.05.

To address a possible role of Nrg1-ErbB axis in mammalian cartilage degradation, we investigated, *in vitro*, its potential to restore the homeostasis of cartilage after mimicking an injury. To that end, we adapted a model of IL1β-induced loss of mature chondrocyte phenotype that we have previously described (Ruiz et al., 2020) (Figure 6A) in which the treatment of chondrocytes with IL1β resulted in an increase of catabolic marker genes which are characteristics of osteoarthritic (OA) chondrocytes. In the present study, we first confirmed the expression of *Nrg1*, *ErbB2* and *ErbB3* mRNA by RT-qPCR in primary control chondrocytes (Figure 6B). Then, we assessed the phenotype of untreated versus degenerated (IL1β) chondrocytes and showed that while NRG1 treatment of chondrocytes did not modify their expression levels of *Nrg1* and its two receptors, IL1β treatment significantly increase the expression levels of *Nrg1* and *ErbB2* (Figure 6B). No effect of IL1β treatment was observed on *ErbB3* (Figure 6B). After demonstrating that chondrocytes could respond to NRG1 both in basal conditions and upon IL1β treatment, we investigated the effect of exogenous NRG1 on the phenotype of OA-like chondrocytes. RT-qPCR analysis revealed that NRG1 treatment prevented the increased expression level of catabolic markers such as *matrix metalloproteinase (Mmp)-13* and *a disintegrin and metalloproteinase motifs 5* (*Adamts5*) induced by IL1β in chondrocytes (Figure 6C). Of note, the catabolic marker *alkaline phosphatase* (*Alpl*) only modestly increased after IL1β treatment. Although this increase did not reached significance, we also observed a tendency to decreased levels in the presence of exogenous NRG1. Altogether, these data reveal a chondroprotective role of NRG1 that prevents the acquisition of OA-like characteristic phenotypes by IL1β-treated chondrocytes. Thus, this study provides the first demonstration that activating the Nrg1/ErbB signaling pathway alleviates IL1β-induced loss of mature mammalian chondrocyte phenotype.

## Discussion

Our study shows that the zebrafish larva is able to regrow a complete MC after surgical amputation of the jaw removing about half of the anterior cartilaginous tissue. The new chondrocytes originate, at least in part, from the chondrocytes remaining intact in the wounded MC that reentry the cell cycle and proliferate to compensate the loss of cartilage. In addition, our data suggest that active proliferation of the chondrocytes located at the border of the wound generate a cluster of new chondrocytes that will reorganize and grow in few days to reform the mass, shape and cell type composition of the original structure.

We then identified for the first time the involvement of the Nrg1/ErbB pathway in the regeneration of cartilage. Indeed, our experiments demonstrated that the expression of the growth factor Nrg1 is specifically induced in the lesioned tissue in close proximity to the cut cartilage, while both the inhibition of ErbB receptors and the specific knockdown of *nrg1* function at the level of the wound epithelium disrupt MC regeneration.

Finally, our *in vitro* experiments indicated that recombinant human NRG1 attenuates IL1β-induced mammalian chondrocyte loss of mature phenotype. Together, these results highlight that targeting the Nrg1/ErbB pathway might be an interesting alternative for enhancing the regenerative potential of cartilage in other species.

Spontaneous capacity to regenerate complex structures exists in zebrafish at adult stages, and skeletal elements are not an exception. Large-scale bone injuries such as the amputation of caudal fin, calvarian bone perforation or jawbone resection are followed by robust regenerative responses, although this is achieved by different processes depending on the nature of the injured bone and the availability of precursors. For instance, fin and skull bones regrow primarily from wound-associated pre-existing osteoblasts that dedifferentiate, proliferate and migrate (Knopf et al., 2011; Geurtzen et al., 2014). Alternatively, zebrafish that have been chemo-genetically depleted in osteoblasts can also regenerate their fin via recruitment of osteoblasts precursors located at the ray joints, precursors that could be replenished by other reservoirs of mesenchymal cells (Singh et al., 2012; Ando et al., 2017). Regeneration of the adult zebrafish jawbone after resections occurs via a cartilaginous intermediate (Wang et al., 2012; Paul and Crump, 2016; Ohgo et al., 2019), whose precursor cells originate in the periosteum and differ from the development stage as they show hybrid cartilage/bone identity (Paul and Crump, 2016; Paul et al., 2016; Ohgo et al., 2019).

Despite extensive work on skeletal regeneration, the regenerative capacity of cartilage itself has been poorly studied in zebrafish. Previous work by Wang et al. (Wang et al., 2012) noted that once the process of jawbone regeneration has completed, the cartilaginous intermediate disappears and the mandibular symphysis is not retained in the regenerated structure. On the contrary, our study performed in zebrafish larvae shows that pre-existing chondrocytes contribute the production of new cartilage via increased cell proliferation triggered by the injury. In addition, after an early step of fusion of the two branches of MC during regeneration, a gap in the continuum of *col2a1* ^+^ maturing cells reappears at the distal tip of MC suggesting that the mandibular symphysis reforms.

These discrepancies might be due to inadequate or missing (re)activation of some signal(s) required to achieve the perfect copy of the loss structure when the lower jaw injury is performed at an adult stage. For example, full limb regeneration in zebrafish is possible at larval stages but almost inexistent in adults (Yoshida et al., 2020) whereas the limb endoskeletal structures can fully regenerate in other fish species (Cuervo et al., 2012).

More recently, studies in zebrafish had shown that zebrafish might be a valuable model to study the genetics of joint cartilage degradation, taking advantages of stable mutants. Indeed, the temporo-mandibular joint has been described as a synovial joint which is sensitive to genetically induced arthritis (Askary et al., 2016; Lawrence et al., 2018) and to post-traumatic degradation in adult (Smeeton et al., 2021). In addition, the latter work demonstrated that articular cartilage in the jaw joint regenerates spontaneously and that *sox10* ^+^ cells are required for this process, which is in line with our results indicating the exhaustion of endogenous *sox10* ^+ high^ cells in the early steps of new chondrocyte production during MC regeneration. Although the molecular signals involved in the regenerative process of the jaw joint have not been addressed in details yet, this model might undoubtedly serve future cartilage research, in particular for the identification of articular cartilage precursors that could be primed to achieve repair in mammals. Meanwhile, our model of complete MC regeneration using young zebrafish larvae that allows extensive screening tests with potential therapeutic drugs appears ideally suited to study the basic mechanisms involved in spontaneous cartilage regeneration.

Consistent with this, we identified for the first time the importance of Nrg1/ErbB pathway in cartilage regeneration in our model. *ErbB3/erbb3b* expression has been described to depend on SoxE transcription factors, respectively Sox10 in cranial NCC in zebrafish (Prasad et al., 2011) and Sox9 in the mammalian heart (Akiyama et al., 2004). High *sox10* expression in larval zebrafish chondrocytes that are not fully mature or re-activation of *sox10* expression in precursors during adult articular cartilage regeneration may account for the production of new chondrocytes in this species upon Nrg1 expression triggered by the injury. We also found in this work the expression of *ErbB3* in murine primary chondrocytes in culture, a cell type that is characterized by Sox9 expression, suggesting that NRG1 could activate ErbB2-ErbB3 downstream pathways in this context.

In stark contrast to zebrafish, mammals are mostly unable to regenerate lost tissues as adults, while some robust regenerative capacities are present only at embryonic or neonatal stages. For instance, the heart and limb bud of the neonatal or embryonic mouse can regrow after amputation (Chan et al., 1991; Porrello et al., 2011). This is thought to occur via the presence of less differentiated cells at these stages that show robust capacity to reactivate proliferation. An exception is the regeneration of the digit tip that also occurs in adult mammals by dedifferentiation of osteoblasts (Storer et al., 2020).

Of note, Nrg1 has been repeatedly involved in different endogenous regenerative processes described in mammalian species (Mahmoud et al., 2015; Bartus et al., 2016). Moreover, addition of exogenous Nrg1 or Nrg1 overexpression have been shown to ameliorate the endogenous repair processes in adult mammals (Hao et al., 2021; Wang et al., 2022a) and to potentiate the regenerative capacity of other factors (Liang et al., 2015; Yoon et al., 2018). Then, Nrg1 appears as an interesting novel candidate factor to design pharmacological based strategy to prevent cartilage degradation, alone or in combination with implantations of engineered scaffolds and therapeutic cells that have been recently developed (Le et al., 2020; Wang et al., 2022b).

The present study, together with previous work from other authors, has also identified the Nrg1/ErbB pathway as a crucial signal required for the regenerative events of distinct structures both in the adult (heart) and larval zebrafish (caudal fin and Meckel’s cartilage), indicating that the Nrg1/ErbB axis may be a universal factor promoting repair and regeneration in different species and contexts.

The role of ErbB signaling during cartilage development and pathology has been poorly investigated so far, although ErbB2 signaling was found to be involved in normal skeletal development (Fisher et al., 2007) and *ErbB4* expression has been described in maturing chondrocytes (Nawachi et al., 2002) in mouse. We detected *ErbB3* and *ErbB2* transcripts in mouse primary chondrocytes in culture, whereas *ErbB4* was found to be absent, as in human chondrocytes (Islam et al., 2001). Although the culture conditions and the age and nature of the source tissue may account for this discrepancy, our *in vitro* data bring new evidences that the Nrg1/ErbB pathway is functional in mammalian cartilage and could be targeted to enhance the repair/regeneration response upon cartilage injury. Notably, it has been described that exogenous NRG1 can induce the proliferation of cardiomyocytes after myocardial infraction by activating Nrg1/ErbB4 pathway (Bersell et al., 2009), and these results have led to several clinical assays to protect or repair the heart (Jabbour et al., 2011a; Jabbour et al., 2011b; Yan and Morgan, 2011; Wadugu and Kuhn, 2012). However, it remains controversial whether the proliferative response in adult mammals would be sufficient to give robust regenerative response, based on other studies that found a decline of this capacity few days after birth aging due to a loss of *ErbB2* expression, the limiting heterodimeric partner of ErbB receptors which controls cardiomyocyte proliferation (D'Uva et al., 2015; D'Uva and Tzahor, 2015; Polizzotti et al., 2015).

New investigations will be needed to precise the effects and mode of action of Nrg1/ErbB signaling in mammalian cartilage repair *in vivo*. Nevertheless, our work provides important insights into the potential role of this signaling pathway in chondrocyte differentiation and regeneration, suggesting that further modulation of Nrg1/ErbB signaling might lead to therapeutic benefit.

## Data Availability

The raw data supporting the conclusion of this article will be made available by the authors, without undue reservation.
