# Supplementary figures and images for: Cartilage regeneration in zebrafish depends on Nrg1/ErbB signaling pathway
Source: Front Cell Dev Biol. 2023 Apr 4;11:1123299. doi: 10.3389/fcell.2023.1123299 (PMC10192884; doi:10.3389/fcell.2023.1123299)

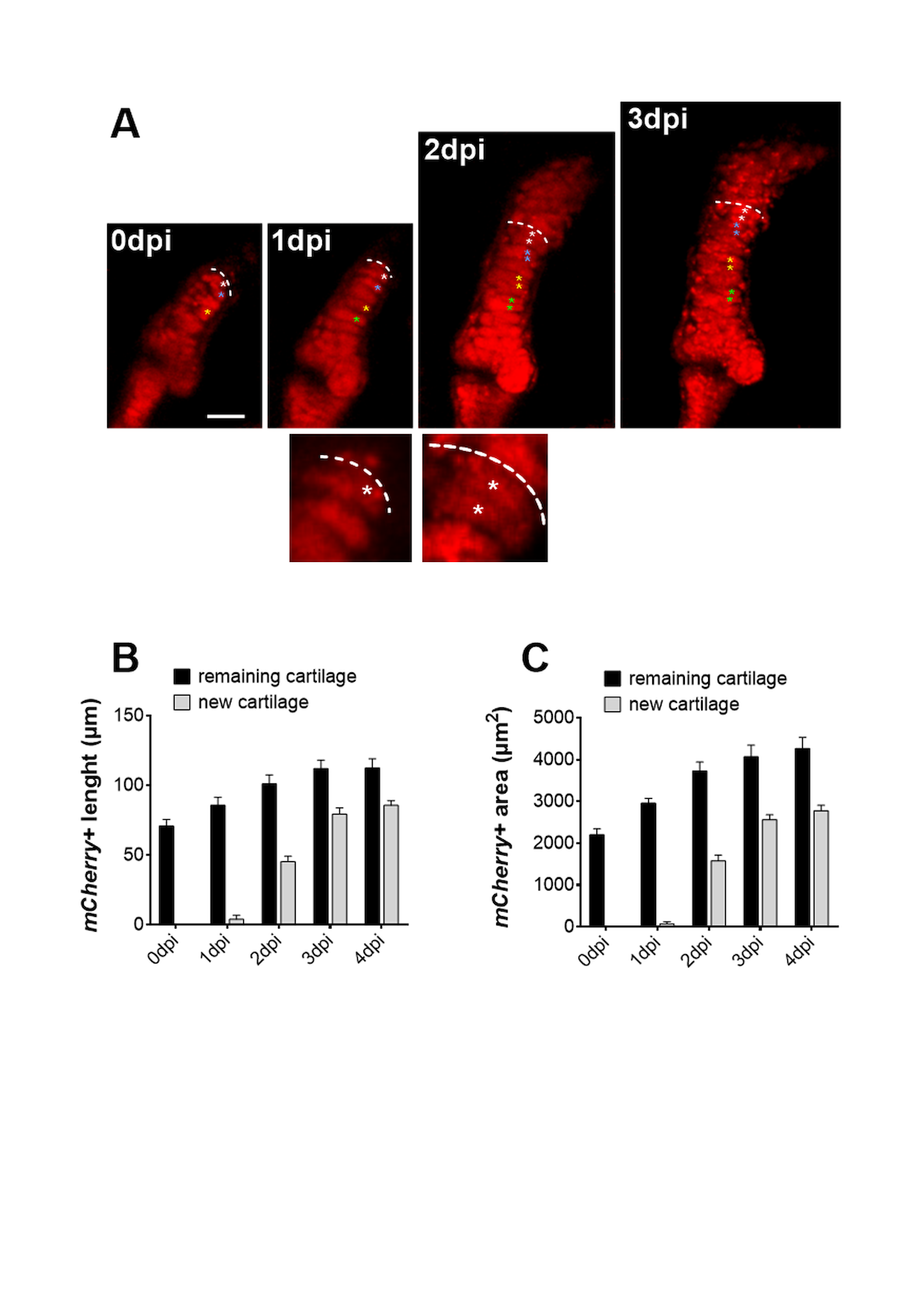

Supplement: Supplementary file 1 [file Image1.TIF]
